# Syndrome‐specific and familial imaging traits in juvenile absence epilepsy

**DOI:** 10.1002/epi.70094

**Published:** 2026-01-13

**Authors:** Fenglai Xiao, Lorenzo Caciagli, Luisa Delazer, Sjoerd Vos, Karin Trimmel, Louis Andre Van Graan, Marine Fleury, Lawrence Binding, Davide Giampiccolo, Dominic Heaney, Sanjeev Rajakulendran, Maria Centeno, Josemir W. Sander, John S. Duncan, Matthias J. Koepp, Britta Wandschneider

**Affiliations:** ^1^ Department of Clinical & Experimental Epilepsy UCL Queen Square Institute of Neurology London UK; ^2^ Chalfont Centre for Epilepsy Chalfont St Peter Bucks UK; ^3^ Department of Neurology Inselspital, Sleep‐Wake‐Epilepsy Center, Bern University Hospital, University of Bern Bern Switzerland; ^4^ Department of Neurology Epilepsy Center, LMU University Hospital, LMU Munich Munich Germany; ^5^ Department of Neurology Medical University of Innsbruck Innsbruck Austria; ^6^ Departments of Computer Science, Medical Physics, and Biomedical Engineering Centre for Medical Image Computing, UCL London UK; ^7^ Neuroradiological Academic Unit, UCL Queen Square Institute of Neurology University College London London UK; ^8^ Western Australia National Imaging Facility University of Western Australia Nedlands Western Australia Australia; ^9^ Department of Neurology Medical University of Vienna Vienna Austria; ^10^ Victor Horsley Department of Neurosurgery National Hospital for Neurology and Neurosurgery London UK; ^11^ Institute of Neurosciences Cleveland Clinic London London UK; ^12^ North Middlesex University Hospital London UK; ^13^ Epilepsy Unit, Department of Neurology Hospital Clínic de Barcelona Barcelona Spain; ^14^ Stichting Epilepsie Instellingen Nederland Heemstede the Netherlands

**Keywords:** endophenotype, magnetic resonance imaging, sensorimotor system, syndrome‐specific

## Abstract

**Objective:**

Juvenile absence epilepsy (JAE) is an idiopathic generalized epilepsy characterized by absences, generalized tonic–clonic seizures, and cognitive difficulties. In contrast to juvenile myoclonic epilepsy (JME), where distinct functional and structural brain alterations are well established, it remains unclear whether comparable changes are identifiable in absence‐predominant syndromes. We aimed to delineate functional and structural correlates of the cognitive profile in people with JAE and to explore potential familial imaging traits.

**Methods:**

We acquired working memory functional magnetic resonance imaging (MRI) and high‐resolution T1‐weighted MRI in 23 individuals with JAE, 18 unaffected siblings, and 28 controls.

**Results:**

Compared with both siblings and controls, patients showed increased motor cortex activation during the attention‐only condition, but relative suppression of motor activity and inadequate default mode network deactivation with increasing working memory demand. Gray matter volume was reduced in sensorimotor regions and in the left inferior and middle frontal gyri in patients. Larger volumes in these frontal regions correlated with better language function. In contrast, increased gray matter volume in the dorsal midcingulate cortex was present in both patients and their siblings relative to controls.

**Significance:**

Our findings in JAE differ from the patterns of functional reorganization reported in JME, indicating that each syndrome involves distinct motor–cognitive pathophysiological mechanisms aligned with its seizure profile. Inferior frontal structural abnormalities likely contribute to the well‐recognized language difficulties in JAE, whereas increased midcingulate gray matter volume may serve as a familial marker linked to attentional vulnerability.

**Key points**

- JAE shows altered motor activation and reduced sensorimotor gray matter, distinct from prior juvenile myoclonic epilepsy work.
- Volume loss in the inferior frontal gyrus may account for language impairment in people with JAE.
- Increased volume of the dorsal midcingulate cortex in people with JAE and their siblings represents a familial trait (endophenotype).
- Impaired default‐mode deactivation during cognitive tasks is a shared feature across idiopathic generalized epilepsies.
- Multimodal imaging combined with cognitive assessments delineates syndrome‐specific mechanisms from familial or generalized epilepsy traits.

## 1 INTRODUCTION

Juvenile absence epilepsy (JAE) is an idiopathic generalized epilepsy syndrome (IGE), along with childhood absence epilepsy (CAE), juvenile myoclonic epilepsy (JME), and epilepsy with generalized tonic–clonic seizures alone.^1^ These syndromes likely represent a neurobiological continuum, given their overlap in age at onset, pathophysiology, treatment response, seizure types, and transitions between syndromes, including cases in which no clear distinction can be made between CAE and JAE.^2^

IGE syndromes have presumed polygenetic underpinnings and high heritability.^1^ Prior functional and structural magnetic resonance imaging (MRI) studies demonstrate that motor system hyperactivity and abnormal segregation of frontal cognitive and motor networks in JME may underlie cognitively triggered myoclonic seizures and, at the same time, impaired working memory and executive function.^3^, ^4^, ^5^ It remains unclear whether such imaging traits are specific to JME or reflect a more general mechanism underlying IGE.

Structural MRI studies in combined cohorts of people with IGE and absences reported reduced gray matter volume in orbitofrontal and temporal cortices^6^ and an association of altered frontotemporal cortical thickness and sulcal depth with intelligence quotient (IQ).^7^ Reduced gray matter volume and surface area were identified in the frontal regions, the anterior cingulate gyrus, and the mesial temporal lobe.^8^ Other work in people with absence seizures described subcortical abnormalities, including smaller thalamic volumes, suggesting aberrant thalamofrontocortical network architecture consistent with other IGE syndromes.^9^, ^10^ Functional imaging in people with absences focused on attention and showed reduced medial frontal cortex activation during a sustained attention task in CAE.^11^ Impaired consciousness during absence seizures is associated with widespread network changes, including decreased frontoparietal activity and increased thalamic involvement, linking behavioral impairment to large‐scale network dysfunction.^12^ Overall, findings indicate that regions involved in generation or propagation of absences overlap closely with networks supporting cognition.

Neuropsychological studies report lower IQ, visuospatial processing, attention, language, and executive function in individuals suffering from absences.^6^ In a recent study, we assessed language functions, including vocabulary, similarities, verbal fluency, and naming, in JAE patients and their unaffected siblings, and found similar impairment in the language domain in both groups.^13^ Comparison of individuals with JAE and JME showed impaired response inhibition in JME but not JAE.^14^

Given the distinct clinical profiles of JAE and JME, notably their differences in seizure predominance, cognitive profiles, and motor involvement, we anticipated that syndrome‐specific alterations would be most evident in neural systems corresponding to these divergences, particularly the motor and sensorimotor networks. In contrast, because attentional and executive impairments are well described across IGEs, and show familial aggregation, we further hypothesized that higher order cognitive networks carry imaging signatures that reflect familial (i.e., present in patients and their siblings) traits rather than syndrome‐specific differences.^6^, ^14^

We hypothesized that (1) people with JAE would show altered motor network recruitment and associated structural changes distinct from healthy controls and those previously observed in JME, whereas (2) alterations within attentional networks might be present in both individuals with JAE and their unaffected siblings, consistent with a potential familial trait (endophenotype). To investigate this, we assessed cognitive functional imaging traits in JAE using a working memory functional MRI (fMRI) paradigm and examined their structural correlates with voxel‐based morphometry (VBM). By relating imaging measures to clinical and cognitive performance, and by comparing individuals with JAE, their unaffected siblings, and healthy controls, we aimed to identify syndrome‐specific imaging markers as well as familial traits.

## 2 MATERIALS AND METHODS

### 2.1 Participants and recruitment

In this prospective, cross‐sectional study, we consecutively enrolled 23 adults with JAE, 18 unaffected siblings of 11 index patients, and 28 healthy controls. Patients were recruited from outpatient clinics at the National Hospital for Neurology and Neurosurgery and the Epilepsy Society, siblings were enrolled through index patients, and controls were recruited from the local community in North‐West London and Chalfont St. Peter, Buckinghamshire, UK between 2013 and 2019.

### 2.2 Standard protocol approvals, registrations, and patient consents

This study was approved by the University College London Institute of Neurology and University College London Hospitals Joint Research Ethics Committee (REC no. 11/LO/0439). All participants provided written informed consent in accordance with the standards of the Declaration of Helsinki.

### 2.3 Neuropsychological tests

All participants performed a comprehensive neuropsychological test battery as described elsewhere.^14^ For data dimensionality reduction, we performed three principal component analyses (PCAs) across all participants as in our previous work,^14^ resulting in four main cognitive domain constructs: (1) executive function (Digit Span Forward, Digit Span Backward, arithmetic, Trail Making Test B‐A, and Stroop interference), (2) memory (Adult Memory and Information Processing Battery: list learning, list recall, design learning, and design recall), (3) language (graded naming test, phonemic fluency, semantic fluency, vocabulary, and similarities), and (4) attention/psychomotor speed (Trail Making Test A, Stroop words, and Stroop colors).

### 2.4 fMRI: Working memory paradigm

We used the same working memory paradigm as in previous studies of JME patients.^4^, ^13^

Participants were presented a sequence of dots appearing on a screen in four possible locations and were instructed to respond by moving a joystick with their right hand. The task involved following the current sequence of dot locations with the joystick in the “0‐Back” control condition, and, during the working memory conditions, following the sequence with a delay of one (1‐Back) or two (2‐Back) dot positions. Each condition was 30 s long and repeated five times in a pseudorandom order, interleaved with 15‐s rest blocks. The entire paradigm, which lasted 11 min 20 s, involved the acquisition of 272 echo planar imaging volumes. Reaction time and click misses were recorded in real time during the scanning.

### 2.5 MRI data acquisition

MRI data were acquired with a 3‐T GE MR750 MRI scanner with a 32‐channel head coil. For fMRI, we employed a 50‐slice gradient echo planar sequence with axial orientation, a 64 × 64 matrix, and an in‐plane voxel size of 3.75 × 3.75 mm. Slice thickness was 2.4 mm with a .1 mm interslice gap. Echo time (TE)/repetition time (TR) for the fMRI scan was 25/2500 ms, and flip angle was 70°. The structural MRI data were acquired using a three‐dimensional T1‐weighted magnetization prepared rapid acquisition gradient echo sequence with TE/TR/inversion time of 3.1/7.4/400 ms and voxel size of 1.0 × 1.0 × 1.0 mm.

### 2.6 fMRI processing and analysis

fMRI data were preprocessed and analyzed using Statistical Parametric Mapping (SPM) 12. Images were realigned, normalized to the Montreal Neurological Institute (MNI) echo planar template, resampled to 3 × 3 × 3 mm voxels, and smoothed with an 8‐mm kernel. A full factorial block design was used for single‐subject analysis, with movement parameters as regressors of no interest. Task conditions were modeled as 30‐s blocks and convolved with the canonical hemodynamic response function. Contrasts compared eachworking memory condition (1‐Back, 2‐Back) to the control task (0‐Back), isolating working memory‐related activation while controlling for motor responses. The 2‐Back‐minus‐1‐Back contrast was used to index higher relative to lower working memory load. Voxelwise contrast estimates included six motion regressors, and scans with mean framewise displacement > .5 mm were excluded.

Group‐level analyses used nonparametric permutation testing with SnPM13 (http://www.nisox.org/Software/SnPM13/) as in our previous work.^15^ One‐sample permutation *t*‐tests were conducted for each task (0‐Back, 1‐Back, 2‐Back) and each contrast (2–Back‐minus‐0‐Back, 1–Back‐minus‐0‐Back, 2‐Back‐minus‐1‐Back). Group differences were assessed with two‐sample permutation *t*‐tests following exploratory permutation‐based *F*‐tests. Age, sex, and missed responses were included as covariates for both task and contrast models.

Exploratory correlation analyses in JAE examined relationships between working memory activation and clinical variables (disease duration, number of generalized tonic–clonic seizures [GTCSs] in the past year, number of antiseizure medications [ASMs]), controlling for task performance using missed responses. Because most errors were omissions, this approach minimized multicollinearity and captured meaningful variability.

Statistical significance was set at two‐tailed *p* < .05, whole‐brain familywise error (FWE)‐corrected using 10 000 permutations. For comparisons not surviving FWE correction, we applied *p* < .001 with a cluster threshold of 20 voxels (10 000 permutations). Figures display whole‐brain group differences at *p* < .001 and 20 voxels for completeness.^16^

### 2.7 Correlation analyses

We correlated task activation patterns across all patients with clinical features including age at onset, epilepsy duration, and number of GTCSs in the past year with permutation‐based multiple regression analyses (10 000 permutations) to address the potential influence of disease burden. Then, we conducted whole‐brain correlation analyses of the task effects (0‐Back, 1‐Back, and 2‐Back) and contrasts (1‐Back‐minus‐0‐Back, 2‐Back‐minus‐0‐Back and 2‐Back‐minus‐1‐Back contrasts) with the PCA factor for memory, executive function, and attention/psychomotor speed in all participants. For these exploratory analyses, we used a statistical threshold of *p* < .001 and 20 voxels across the whole brain.^17^

### 2.8 Structural MRI processing and analysis

For structural MRI data analysis, we performed VBM using SPM12 (https://www.fil.ion.ucl.ac.uk/spm/). Images underwent visual quality checks, tissue segmentation, and normalization to MNI space with modulation to account for intracranial volume. Deformation fields were estimated using the DARTEL algorithm, and spatially normalized, Jacobian‐scaled gray matter images were smoothed with a 10‐mm kernel at 1.5‐mm voxel size.

We conducted independent *t*‐tests comparing patients with healthy controls and siblings with healthy controls, following exploratory permutation‐based *F*‐tests. Whole‐brain correlation analyses were performed with clinical variables (disease duration, GTCSs in the past year, number of ASMs) and PCA factors for memory, executive function, attention/psychomotor speed, and language. Intracranial volume, sex, and age were included as covariates, except in disease‐duration analyses (sex only). Statistical significance was set at two‐tailed *p* < .05, FWE‐corrected; exploratory results at *p* < .001, *k* > 200 are reported when FWE correction was not survived.^18^

### 2.9 Statistical analysis of demographic and clinical data

Data were analyzed using IBM SPSS v.28 and R 4.0.3. For comparisons of demographic and clinical data, we used analysis of variance, Kruskal–Wallis test, and Fisher exact test for continuous parametric, nonparametric, and categorical data, respectively; Bonferroni‐corrected post hoc tests were applied for multiple comparisons. Performance data of the fMRI task (number of joystick moves, accuracy, and reaction time) were compared with Kruskal–Wallis tests adjusting for age and sex followed by Bonferroni‐corrected post hoc tests in the case of significant results.

## 3 RESULTS

### 3.1 Demographic, clinical, and neuropsychological data

All 23 individuals experienced absence seizures, 19 (83%) also had GTCSs, and three (13%) reported infrequent myoclonus compatible with JAE; six patients (26%) had been seizure‐free for at least 1 year prior to the investigation. Individuals' age at onset was typical for JAE,^1^ with onset in late childhood to early puberty (median = 12 years), except for seven patients with onset at <8 years of age who may have either had an unusually early age at onset or progressed from CAE to JAE. In these patients with earlier onset, the diagnosis of JAE was based on other syndrome characteristics considered more typical of JAE than CAE, including the occurrence of GTCSs (five of seven patients) and the nondaily frequency of absences.^19^

Routine electroencephalograms (EEGs) showed characteristic interictal 3–4‐Hz generalized spike–wave discharges. No patients' siblings had seizures, except one individual who experienced a single provoked GTCS following head trauma in a motor vehicle accident.

We normalized the drug load as previously described.^20^ Demographic and clinical data are presented in Table 1.

**TABLE 1:** Demographic data, clinical characteristics, and questionnaires.

Group	JAE, *n* = 23	SIB, *n* = 18	CTR, *n* = 28	Test statistics	*p*	Post hoc tests (Bonferroni corrected)
Age, years, median, (IQR), (range)	24.9 (7.0) (16.5–40.0)	25.7 (7.7) (16.5–40.5)	26.3 (5.7) (16.5–43.5)	*F* = .30	.74	
Sex, F/M	16/7	6/12	21/7	FET = 8.9	.01*	JAE vs. SIB .03* JAE vs. CTR .76 SIB vs. CTR .007*
Education, years, median (IQR)	13.0 (3.5)	13.0 (1.5)	16.0 (3.25)	*H* = 16.8	<.001*	JAE vs. SIB 1 JAE vs. CTR .001* SIB vs. CTR .002*
Performance, 0‐Back, % correct responses, mean (SD)	92.6 (9.4)	87.5 (18.2)	94.9 (10.6)	*F* = 1.8	.18	
Performance, 1‐Back, % correct responses, mean (SD)	72.3 (25.0)	79.8 (20.6)	84.3 (14.3)	*F* = 2.1	.13	
Performance 2‐Back, % correct responses, mean (SD)	48.8 (26.5)	68.2 (17.0)	75.6 (22.2)	*F* = 6.9	.002*	JAE vs. SIB .14 JAE vs. CTR .002* SIB vs. CTR .46
Missed responses during WM tasks, *n*, median (IQR)	39.5 (50.5)	20.0 (7.5)	16.0 (14.0)	*H* = 14.4	<.001*	JAE vs. SIB .003 JAE vs. CTR <.001 SIB vs. CTR .79
Missed responses during 0‐Back, median (IQR)	4.0 (6.5)	3.0 (16.0)	1.0 (5.0)	*H* = 3.2	.202	
Missed responses during 1‐Back, median (IQR)	9.5 (32.0)	10.0 (26.5)	7.0 (13.0)	*H* = 2.1	.355	
Missed responses during 2‐Back, median (IQR)	39.0 (28.5)	20.0 (19.5)	14.0 (21.0)	*H* = 10.4	.005*	JAE vs. SIB .015* JAE vs. CTR .003* SIB vs. CTR .29
Reaction time, ms, median (IQR)	811.1 (215.1)	742.9 (267.7)	764.0 (192.0)	*H* = 2.9	.229	
Disease duration, years, median (IQR)	11.3 (11.0)					
Age at onset, years, median (IQR)	12.0 (5.5)					
GTCSs, monthly frequency, median (IQR)	.33 (.77)					
Number of ASMs tried, median (IQR)	3.0 (1.0)					
Drug dosage score, mean (SD)^a^	7.2 (5.1)					

Abbreviations: ASM, antiseizure medication; CTR, controls; F, female; FET, Fisher exact test statistic; GTCS, generalized tonic–clonic seizure; IQR, interquartile range; JAE, juvenile absence epilepsy; M, male; SIB, unaffected siblings of JAE patients; WM, working memory.

^a^ The medication load is normalized for each ASM according to British National Formulary guidelines (https://bnf.nice.org.uk/drug/).

* Statistically significant.

The detailed PCA of neuropsychological data was described in our previous study.^14^ We verified that the first principal component (PC1) had an eigenvalue > 1: (1) executive function (eigenvalue = 1.96, explained variance = 49.0%), (2) memory (eigenvalue = 2.37; explained variance = 59.1%), (3) language (eigenvalue = 2.63, explained variance = 52.5%), and (4) attention/psychomotor speed (eigenvalue = 1.85, explained variance = 61.7%).

### 3.2 fMRI working memory paradigm: Group comparisons

fMRI data were available for 20 people with JAE, 18 siblings, and 27 healthy controls. People with JAE had significantly more missed joystick responses during the 2‐Back condition than siblings and controls, whereas missed responses were comparable across groups for the 0‐Back and 1‐Back conditions (Table 1). There was no difference in response time between groups (Table 1).

**FIGURE 1:**
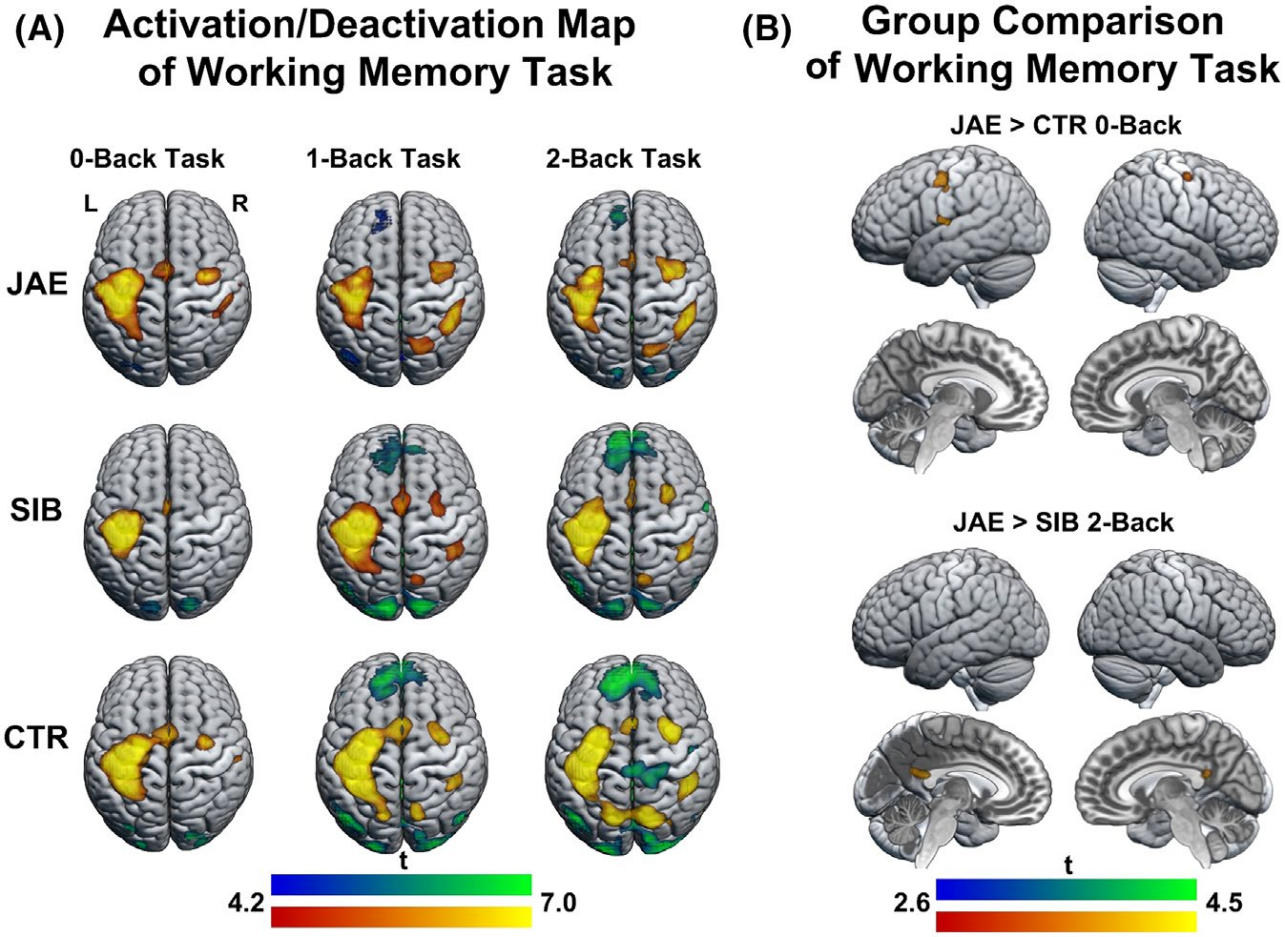
Working memory task conditions: group‐level effects and group comparisons. Brain renderings and sections show voxel‐based activation (warm colors) and deactivation (cold colors) during working memory task conditions (0‐Back, 1‐Back, 2‐Back) in people with juvenile absence epilepsy (JAE), siblings (SIB), and healthy controls (CTR), as derived from one‐sample *t*‐tests. (A) Main task effects in each group: motor cortex and supplementary motor area activation for 0‐Back, motor activation and bilateral medial frontal and parietal deactivation for 1‐Back and 2‐Back. (B) People with JAE had higher activation in the bilateral precentral gyri compared to healthy controls during 0‐Back and less deactivation of the posterior cingulate cortex compared with siblings during 2‐Back. The maps are displayed at an uncorrected threshold (*p* < .001, cluster extent threshold of 20 voxels).

Analyses of working memory task contrasts revealed progressively greater bilateral frontoparietal activation as task load increased, accompanied by corresponding increases in deactivation of default mode regions, including the medial parietal and medial frontal cortices in all groups (Figure 1A).

In group comparisons, patients showed hyperactivation of the bilateral precentral gyrus (left: *p* < .001, right: *p* < .001) and the left superior temporal lobe (*p* < .001) during the 0‐Back condition compared to healthy controls. During the 2‐Back condition, there was reduced deactivation in the posterior cingulate cortex (PCC) in people with JAE compared to their siblings (*p* < .001; Figure 1B). No significant group differences were found in other comparisons of the task conditions.

**FIGURE 2:**
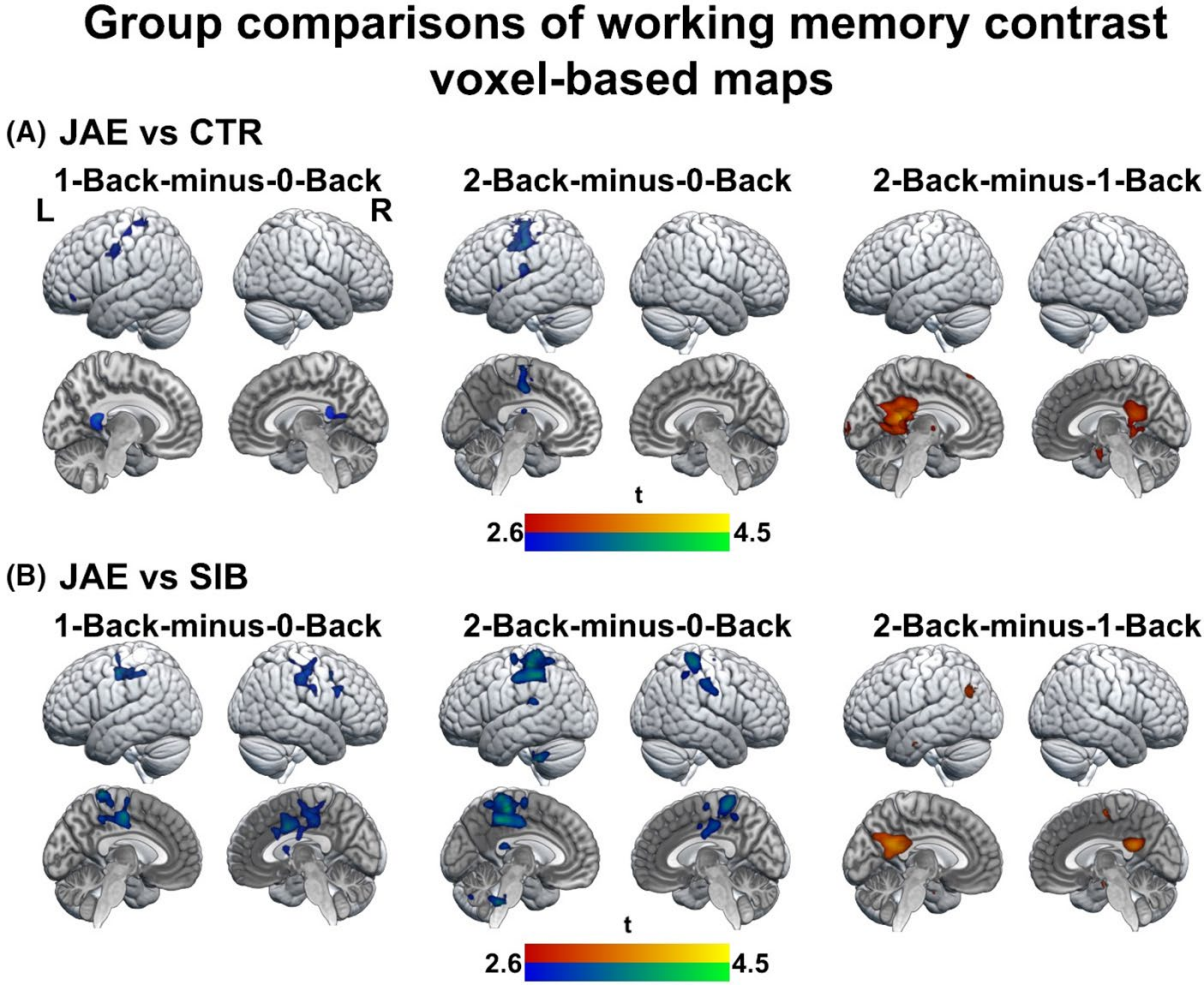
Working memory task contrasts: group comparisons. Brain renderings and sections show voxel‐based activation (warm colors) and deactivation (cold colors) differences for task contrasts among people with juvenile absence epilepsy (JAE), unaffected siblings (SIB), and healthy controls (CTR), as derived from full factorial analyses. (A) People with JAE exhibited decreased activity in the motor regions in the 1‐Back‐minus‐0‐Back contrast and in the 2‐Back‐minus‐0‐Back contrast compared to healthy controls. When contrasting high versus low working memory demands (2‐Back‐minus‐1‐Back), people with JAE showed less deactivation in the posterior cingulate cortex. (B) Comparing people with JAE with siblings demonstrated similar effects. The maps are displayed at a corrected threshold (*p* < .001, 20 voxels).

Working memory task contrasts of each group are shown in Figure S1. Group comparisons of the working memory task contrasts 1‐Back‐minus‐0‐Back and 2‐Back‐minus‐0‐Back demonstrated relatively reduced activation of motor areas in patients compared to controls and siblings (*p* < .001, respectively; Figure 2, Table S1). When contrasting high versus low working memory demand (2‐Back‐minus‐1‐Back), patients showed less deactivation of the PCC, part of the default mode network (DMN), than healthy controls and siblings (*p* < .001, *p*_FWE_ < .05; Figure 2, Table S1). No significant differences were found between siblings and healthy controls.

### 3.3 Correlation of fMRI maps with cognitive performance and clinical characteristics

**FIGURE 3:**
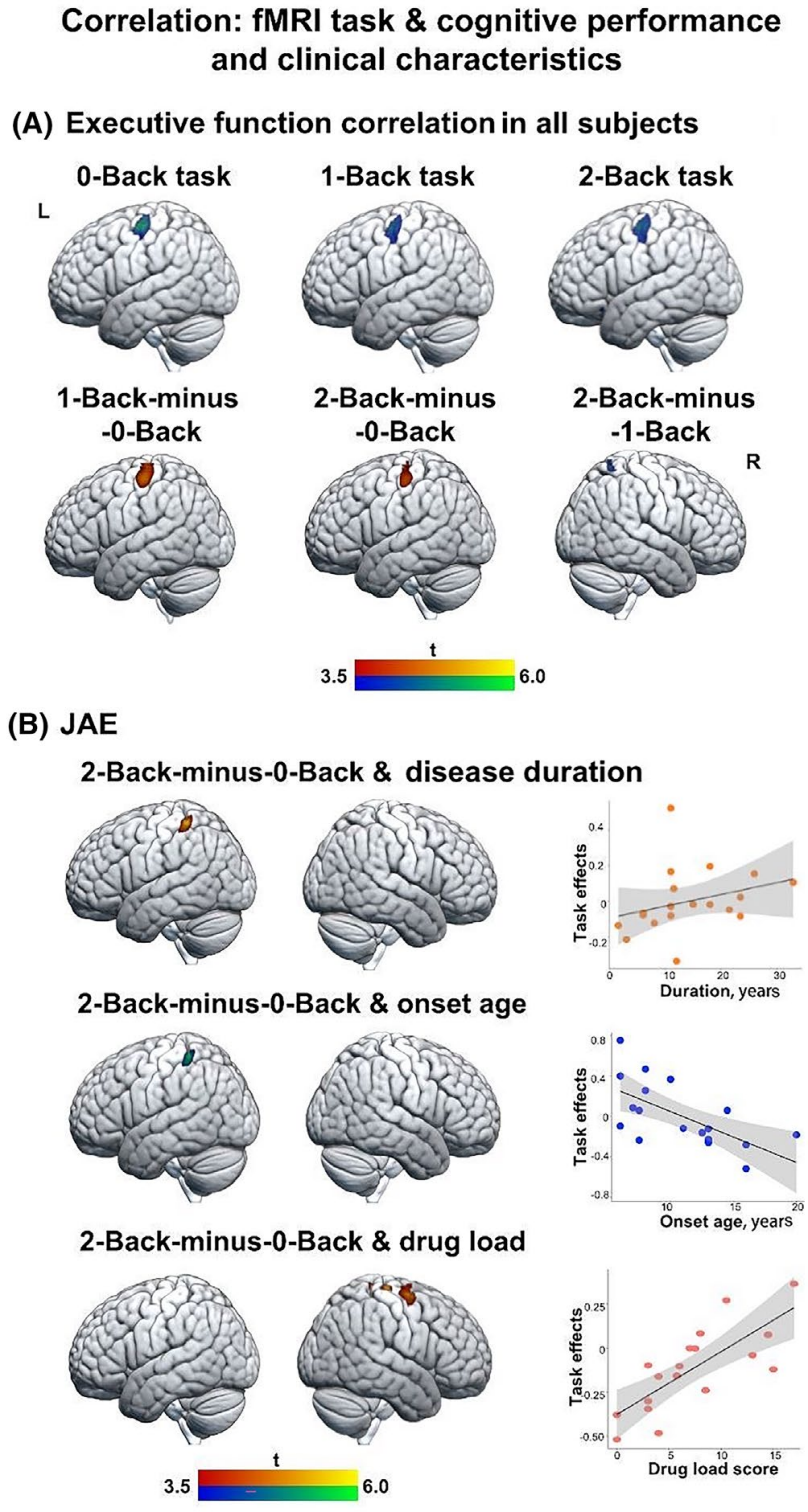
Correlation analyses of working memory functional magnetic resonance imaging (fMRI) with cognitive performance and clinical characteristics. Brain renderings show statistical maps of nonparametric multiple regressions probing associations between working memory fMRI and the executive function principal component analysis (PC1). Warm color scales refer to positive associations, whereas cold color scales refer to negative associations. (A) In all participants, the PC1 correlated with higher activations for both 1‐Back‐minus‐0‐Back and 2‐Back‐minus‐0‐Back in the left motor regions. (B) In people with juvenile absence epilepsy (JAE), longer disease duration and earlier age at onset were associated with higher activations for the 2‐Back‐minus‐0‐Back contrast (*p* < .001, 20 voxels); higher dosage score was positively correlated with higher activations for the 2‐Back‐minus‐0‐Back contrast (*p* < .001, 20 voxels). Higher drug load score was related to higher activation in the right paracentral gyrus and supplementary motor area. The maps are displayed at an uncorrected threshold (*p* < .001, 20 voxels).

In all participants, the executive function PC, with increasing values indicating better executive function performance, correlated with higher activation in the left precentral gyrus for the 1‐Back‐minus‐0‐Back and 2‐Back‐minus‐0‐Back working memory contrasts (*p* < .001), and in the right superior parietal lobe for the 2‐Back‐minus‐1‐Back contrast (Figure 3A, Table S2). Correlation analyses of the memory and attention PC with task‐related activity were not significant.

In patients, longer disease duration and earlier age at onset were associated with higher activation of somatosensory regions for the 2‐Back‐minus‐0‐Back working memory contrast (*p* < .001). A higher drug dose score was associated with higher activation in the right paracentral gyrus and supplementary motor area (SMA; *p* < .001; Figure 3B, Table S2). We observed no correlations of task‐related activation with the number of GTCSs in the year prior to the study or the number of ASMs.

### 3.4 Structural MRI: Group comparisons

**FIGURE 4:**
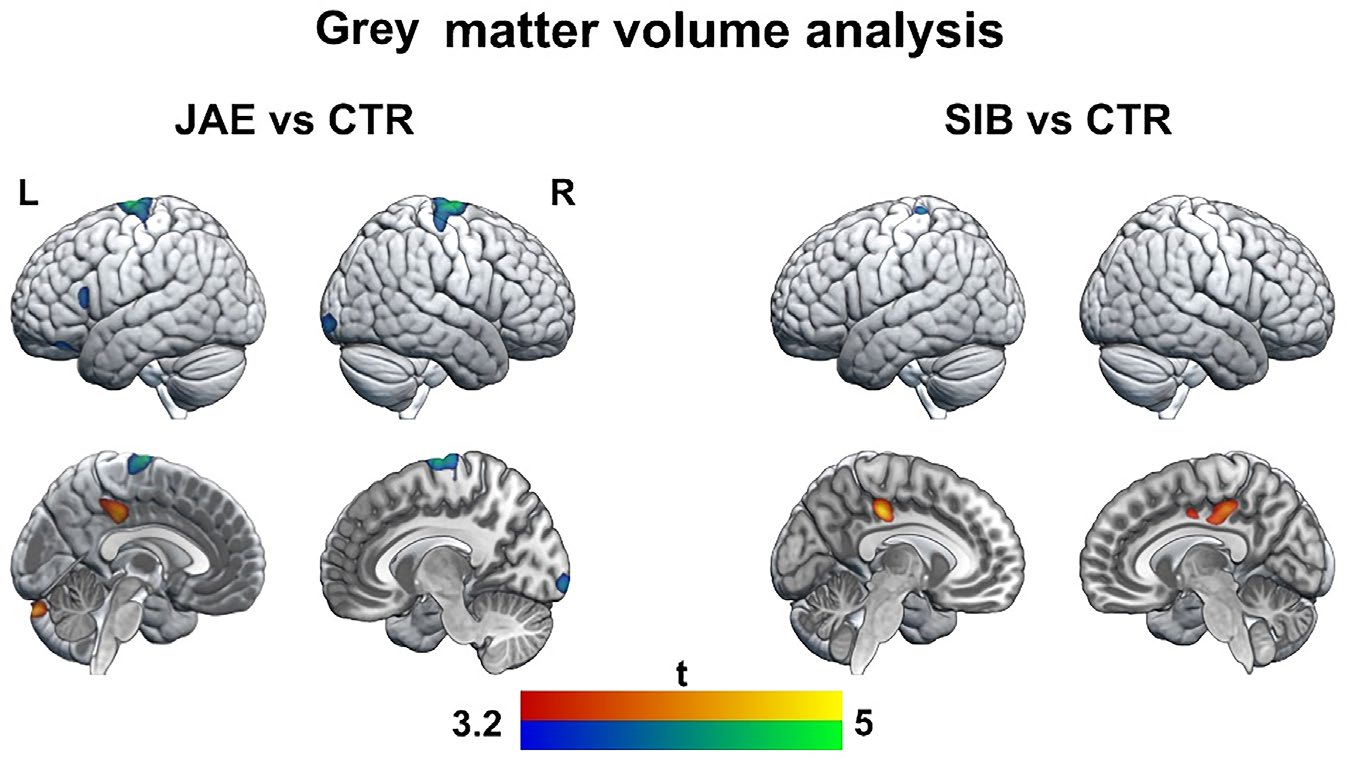
Gray matter volume analysis: group differences. Brain renderings and sections show statistical maps of voxel‐based analysis of structural magnetic resonance imaging among people with juvenile absence epilepsy (JAE), siblings (SIB), and controls (CTR), as derived from independent two‐tailed *t*‐tests. Warm color scales refer to positive associations; cold color scales refer to negative associations. Compared to controls, people with JAE showed reduced gray matter volumes in bilateral precentral gyrus and paracentral lobule (motor regions), left inferior frontal gyrus, and right medial occipital cortex as well increased gray matter volume in the left dorsal middle cingulate cortex (dMCC). In siblings, we found no decreased brain volumes compared to controls but increased gray matter volumes in the dMCC bilaterally, similar to those seen in people with JAE. The maps are shown at an uncorrected threshold (*p* < .001, 200 voxels).

Structural MRI was available for 23 people with JAE, 17 siblings, and 28 healthy controls. Patients had reduced gray matter volumes in bilateral precentral gyrus and paracentral lobule (motor regions), left inferior frontal gyrus (IFG), and right medial occipital cortex than controls. There were increased gray matter volumes in JAE in the left dorsal middle cingulate cortex. In siblings, we found no reduced gray matter volumes compared to controls but increased gray matter volumes in the dorsal middle cingulate cortex bilaterally, similar to those seen in people with JAE (Figure 4, Table S1; *p* < .001).

### 3.5 Correlation of structural MRI findings with clinical data and neuropsychological performance

**FIGURE 5:**
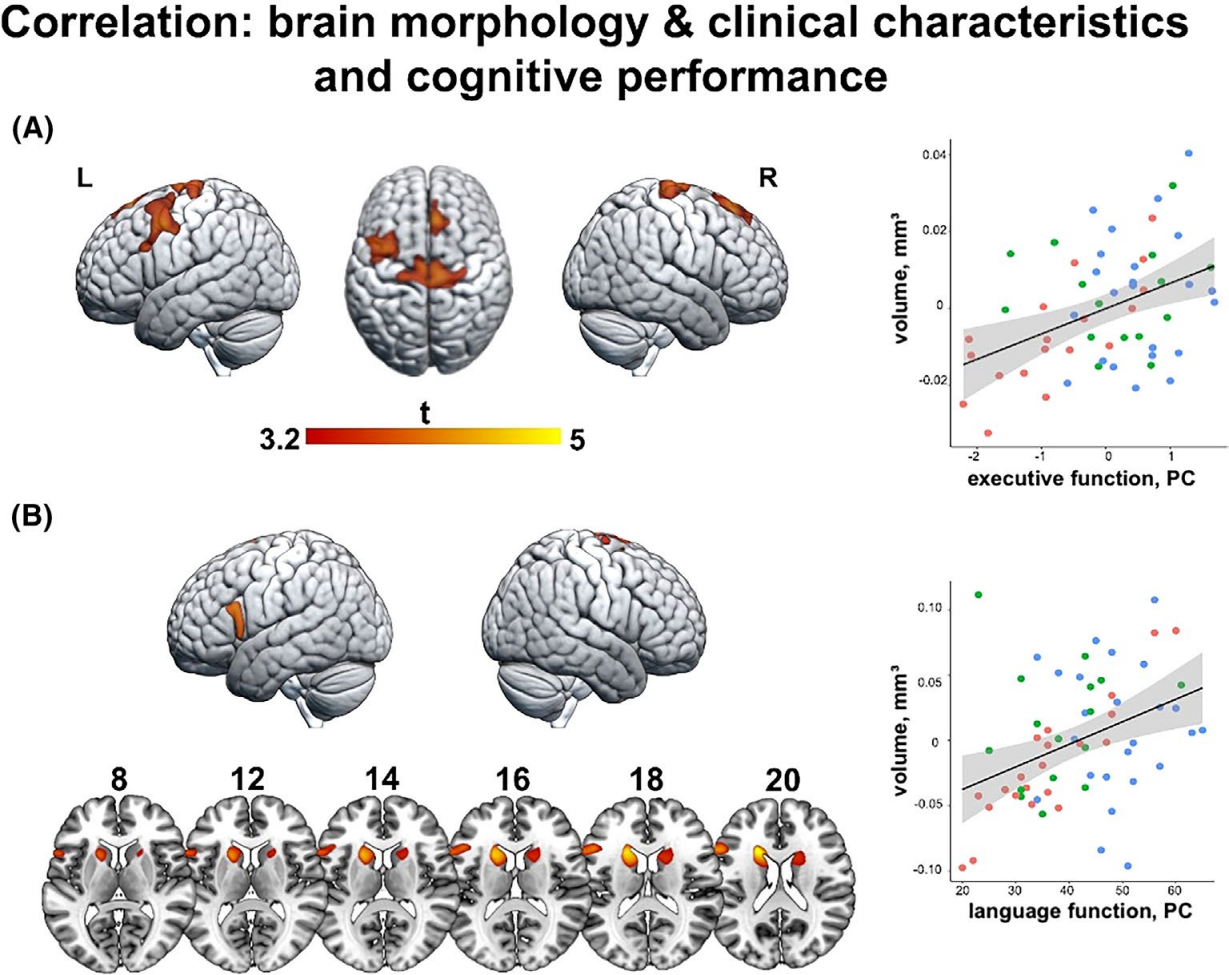
Correlation of gray matter volume with cognitive performance. Brain renderings and sections display statistical maps of nonparametric multiple regressions probing associations between brain volume and cognitive performance in all participants (red dots = juvenile absence epilepsy; green dots = siblings; blue dots = healthy controls.). Warm color scales refer to positive associations. (A) Better executive functions (principal component [PC]) correlated with increased volumes in the sensorimotor regions, right superior frontal gyrus, and left cuneus. (B) Better language function (PC) was associated with higher gray matter volumes in the left middle and inferior frontal gyri right supplementary motor area and bilateral caudate nuclei. The maps are shown at an uncorrected threshold (*p* < .001 uncorrected, 200 voxels).

Better executive function (PC1) performance correlated with greater volumes of bilateral motor regions, right superior frontal gyrus, and left cuneus (all *p* < .001; Figure 5A, Table S2). Better language function (PC1) was associated with greater gray matter volumes in the left middle and IFG, right SMA, and bilateral caudate nucleus (all *p* < .001; Figure 5B, Table S2). Whole‐brain correlation analyses showed no significant associations of patterns of gray matter volume abnormalities in patients with disease duration, age at onset, or frequency of GTCSs in the past year.

## 4 DISCUSSION

In this study, we hypothesized that syndrome‐specific alterations in JAE would be most evident in motor and cognitive‐control networks, reflecting the distinctive behavioral phenotype of absence seizures, whereas familial traits (endophenotypes) would manifest in higher order networks involved more broadly in attention and executive control. Our findings support this framework by demonstrating both distinct structural–functional signatures in motor systems in individuals with JAE and shared familial features in cingulate‐salience regions in patients and their siblings.

### 4.1 Increased motor activity and reduced gray matter volumes in the sensorimotor region: A syndrome‐specific structural–functional profile in JAE

People with JAE showed increased motor cortex activation during the attentional control condition, but relatively less motor activation as working memory load increased. Behaviorally, these differences were accompanied by poorer 2‐Back performance. Collectively, these findings suggest an abnormal recruitment pattern, in which individuals with JAE overengage the motor system under minimal cognitive demand yet fail to sustain appropriate activation with increasing task difficulty, consistent with the characteristic attentional impairment described in absence epilepsies (AE).^19^

VBM revealed reduced gray matter volume in sensorimotor regions in JAE, consistent with previous work in JAE and^8^ JME^21^ and in larger IGE cohorts.^22^ The novelty of our study lies in demonstrating that both functional activation profiles and structural abnormalities converge within motor regions. Brain–behavior correlations further support this analysis; better executive performance was associated with greater motor activation and higher gray matter volume within motor cortex.

These effects mapped onto regions comprising the somatocognitive action network (SCAN), which comprises the SMA and other areas functionally connected to the dorsal anterior cingulate, parietal areas, and sensory divisions of the insula integrating motor output with action planning and cognitive control.^23^, ^24^ Damage or inefficient recruitment within the SCAN provides a unified explanation for the observed motor and cognitive abnormalities, and aligns with broader models of IGE network dysfunction, including involvement of the SMA in JME,^4^, ^13^ and SCAN involvement in deep brain stimulation‐responsive epilepsy networks.^25^ Interestingly, our findings are also consistent with those of a task‐related EEG‐fMRI study in a mixed AE group, which, in retrospect,^12^ also appears to involve SCAN areas. Clinically, disease load (younger onset, longer duration) was associated with increased motor activation, whereas higher ASM doses correlated with more normalized activation patterns, as seen previously with valproate in JME.^4^ This suggests that structural abnormalities may be relatively static, whereas functional responses remain dynamic and treatment modulated.

### 4.2 Contrasting motor profiles across IGE syndromes

Our findings of motor hyperactivity under low cognitive demands and reduced motor activity during higher task demands stand in clear contrast to the well‐described motor hyperexcitability in JME during the same working memory task.^4^ Prior studies in JME^4^, ^5^, ^13^ show robust motor hyperactivation and prefrontal–motor hyperconnectivity.^3^, ^4^, ^5^ Together with transcranial magnetic stimulation studies showing increased corticospinal excitability,^26^ our fMRI data support a pathophysiological model in which cognitive load can trigger myoclonic seizures, the clinical hallmark of JME. In contrast, the opposite activation patterns we observed in JAE, with reduced motor system engagement with increasing cognitive demands, mirrors the characteristic motor arrest of absence seizures. Thus, the two syndromes may represent opposite ends of a shared motor–cognitive network dysfunction, with JME expressing the hyperkinetic extreme (myoclonus) with unimpaired consciousness, and JAE the hypokinetic extreme (behavioral arrest) with impaired consciousness.

These opposing functional signatures, despite both syndromes showing gray matter loss within motor regions, provide compelling evidence for syndrome‐specific reorganization of motor networks across the IGE spectrum. Structural abnormalities appear to be a common substrate,^22^ but the direction and dynamics of motor system engagement under cognitive load differentiate syndromes dominated by motor hyperexcitability from those characterized by movement stillness, or only subtle eye‐blinking, during absences.

### 4.3 Dorsal midcingulate enlargement: A potential endophenotype?

Patients with JAE showed increased gray matter volume in the dorsal midcingulate cortex, part of the salience network,^27^ which governs switching between the DMN and task‐positive networks.^28^ This enlargement was also present in their unaffected siblings. Alterations in these areas may predispose individuals to attentional inefficiency, even in the absence of overt cognitive impairment in siblings. Dysfunction of the salience network is linked to disruption of attention and awareness circuits, core features of absence seizures^11^ with impaired attention representing the hallmark cognitive finding in AE.^6^

Given that IGEs are genetically determined neurodevelopmental conditions, increased gray matter volume in the dorsal midcingulate cortex likely reflects genetic influences resulting in impaired neuronal pruning,^29^ rather than disease activity or treatment effects. The presence of this structural feature in both groups is therefore consistent with an endophenotype, helping to differentiate inherited neurodevelopmental differences from syndrome‐specific functional traits.

### 4.4 IFG alterations and language dysfunction: A syndrome‐linked structural substrate

Reduced gray matter volume in the left IFG provides a plausible structural substrate for language impairment in JAE, a well‐recognized clinical feature.^30^, ^31^ Structural correlations revealed that better language performance was associated with greater gray matter volume in the left middle and IFGs, SMA, and caudate nuclei, regions with established roles in language processing and corticostriatal control of executive and linguistic functions.^32^ Thus, inferior frontal volume loss aligns with the syndrome's characteristic cognitive phenotype, supporting the notion that JAE has distinct structural underpinnings affecting language‐related circuits.

### 4.5 Regionally impaired DMN deactivation: A marker for network dysfunction across epilepsies

During the higher demand working memory condition (2‐Back and 2‐Back‐minus‐1‐Back analyses), people with IGE and absences showed failure to appropriately deactivate the PCC compared to both their unaffected siblings and controls. The PCC is a core hub of the DMN, a system engaged during mind wandering, introspection, and internally driven cognition.^33^ It also plays a well‐established pathophysiological role in absence seizures.^12^ In an EEG‐fMRI study capturing absence events, abnormal DMN activity preceded reduced sensorimotor input and impaired engagement of a broad task‐positive network^12^ partly overlapping with the dorsal attention network.^34^ Deactivation of DMN regions during cognitive tasks is normally required to reallocate neural resources toward task‐relevant networks.^35^ Thus, impaired DMN suppression in people with absences likely reflects inefficient resource allocation and may constitute an additional neural substrate for the cognitive vulnerabilities observed in JAE.^14^ Unlike the sensorimotor hypoactivation pattern, however, abnormal DMN deactivation is not specific to JAE. Prior work demonstrated altered DMN dynamics not only in temporal lobe epilepsy with failure to deactivate the ipsilateral hippocampus and in the motor cortex in JME,^4^, ^15^ but also across a wide range of psychiatric disorders, including autism^36^ and schizophrenia.^37^ Similar effects have been observed in pharmacological–fMRI studies examining the impact of ASMs.^20^, ^38^, ^39^, ^40^, ^41^, ^42^ Taken together, DMN dysfunction may represent a shared marker of disrupted large‐scale network organization, whether driven by epilepsy syndrome, medication effects, or other brain disorders, representing a plausible common pathway underlying cognitive impairment across multiple conditions.

### 4.6 Limitations

Participants were recruited from a tertiary adult epilepsy center, and most had active epilepsy, making the cohort somewhat atypical for JAE, which commonly responds well to treatment. Clinical overlap among IGEs is well recognized, and in the absence of definitive biomarkers, diagnosis remains largely clinical. Although all participants met diagnostic criteria for JAE at the time of scanning, some may represent CAE evolving into JAE, or unusually early onset cases. IGE is a dynamic rather than static condition. In a recent multicenter study of longitudinal MRI in newly diagnosed and chronic, medically refractory IGE, we observed cortical changes exclusively in people with chronic IGE.^43^

Despite being the largest study of well‐characterized individuals with JAE, the sample size remains modest and may limit generalizability. The lack of simultaneous EEG during fMRI and neuropsychological assessments precludes evaluation of the influence of subclinical epileptiform activity. Nevertheless, real‐time task monitoring revealed no prolonged response pauses or group differences in reaction times during working memory tasks. Finally, JME was not included as a comparator group, limiting the ability to definitively infer syndrome specificity.

## 5 CONCLUSIONS

Taken together, our findings support a coherent pathophysiological framework encompassing syndrome‐specific, familial, and IGE‐wide traits. In JAE, syndrome‐specific features include abnormal motor system recruitment under cognitive load, reduced motor system gray matter, and structural alterations of the IFG, which align with the attentional and language difficulties characteristic of the syndrome. Familial traits shared with unaffected siblings center on increased dorsal midcingulate volume within the salience network, suggesting a heritable alteration in systems involved in cognitive control and interoceptive signaling. Across the broader IGE spectrum, impaired DMN deactivation during demanding working memory conditions emerges as a shared functional marker of inefficient resource allocation and possible interference of epileptic activity.

This integrated structural–functional profile highlights the utility of combining multimodal imaging with cognitive phenotyping to dissociate syndrome‐specific mechanisms from familial or broader generalized‐epilepsy network traits.

## AUTHOR CONTRIBUTIONS

Lorenzo Caciagli, Matthias J. Koepp, and Britta Wandschneider contributed to the conception and design of the study. Fenglai Xiao, Lorenzo Caciagli, Luisa Delazer, Sjoerd Vos, Karin Trimmel, Louis Andre Van Graan, Marine Fleury, Lawrence Binding, Davide Giampiccolo, Dominic Heaney, Maria Centeno, Sanjeev Rajakulendran, Josemir W. Sander, John S. Duncan, Matthias J. Koepp, and Britta Wandschneider contributed to the acquisition and analysis of data. Fenglai Xiao, Lorenzo Caciagli, Luisa Delazer, Matthias J. Koepp, and Britta Wandschneider were involved in drafting the manuscript and figures. All authors reviewed and approved the final draft.

## FUNDING INFORMATION

This study was funded by a Henry Smith Charity (20133416) grant awarded to M.J.K. and B.W., by a Wellcome Trust grant (079474) awarded to M.J.K., and by a Brain Research UK scholarship (14181) awarded to L.C. B.W. received salary support from the German Research Foundation (WA3135/1‐1) and acknowledges support from the MRC (MR/T005335/1). F.X. is supported by the Wellcome Trust (221934/Z/20/Z) and the Newton International Fellowship of the Academy of Medical Sciences and the Newton Fund (NIF\R5\264). L.D. received financial support from the European Academy of Neurology, Michael Foundation, and Erasmus Plus. K.T. was supported by scholarships from the European Academy of Neurology and the Austrian Neurology Society. J.S.D. acknowledges support from the NIHR and Wellcome Trust (218380). J.W.S. receives support from the Dr. Marvin Weil Epilepsy Research Fund and the Christelijke Verenigingvoor de Verpleging van Lijdersaan Epilepsie, the Netherlands. M.F. is supported by the Epilepsy Society. The authors acknowledge the facilities and scientific and technical assistance of the National Imaging Facility, a National Collaborative Research Infrastructure Strategy (NCRIS) capability, at the Centre for Microscopy, Characterization, and Analysis, University of Western Australia.

## CONFLICT OF INTEREST STATEMENT

None of the authors has any conflict of interest to disclose. We confirm that we have read the Journal's position on issues involved in ethical publication and affirm that this report is consistent with those guidelines.

## Supporting information


**Table S1.** Group comparisons of functional and structural analysis.
**Table S2.** Correlation analyses of working memory functional magnetic resonance imaging and structural voxel‐based analysis.
**Figure S1.** Working memory task contrasts for each group.

## Data Availability

Anonymized data are available from the corresponding author upon reasonable request from any qualified person.

## References

[1] Hirsch E , French J , Scheffer IE , Bogacz A , Alsaadi T , Sperling MR , et al. ILAE definition of the idiopathic generalized epilepsy syndromes: position statement by the ILAE Task Force on Nosology and Definitions. Epilepsia. 2022;63(6):1475–1499. 10.1111/epi.17236 35503716

[2] Vorderwülbecke BJ , Wandschneider B , Weber Y , Holtkamp M . Genetic generalized epilepsies in adults—challenging assumptions and dogmas. Nat Rev Neurol. 2022;18(2):71–83. 10.1038/s41582-021-00583-9 34837042

[3] Vollmar C , O'Muircheartaigh J , Symms MR , Barker GJ , Thompson P , Kumari V , et al. Altered microstructural connectivity in juvenile myoclonic epilepsy the missing link. Neurology. 2012;78(20):1555–1559. 10.1212/WNL.0b013e3182563b44 22551729 PMC3348847

[4] Vollmar C , O'Muircheartaigh J , Barker GJ , Symms MR , Thompson P , Kumari V , et al. Motor system hyperconnectivity in juvenile myoclonic epilepsy: a cognitive functional magnetic resonance imaging study. Brain. 2011;134(6):1710–1719. 10.1093/brain/awr098 21616969 PMC3102244

[5] Caciagli L , Wandschneider B , Centeno M , Vollmar C , Vos SB , Trimmel K , et al. Motor hyperactivation during cognitive tasks: an endophenotype of juvenile myoclonic epilepsy. Epilepsia. 2020;61(7):1438–1452. 10.1111/epi.16575 32584424 PMC7681252

[6] Ratcliffe C , Wandschneider B , Baxendale S , Thompson P , Koepp MJ , Caciagli L . Cognitive function in genetic generalized epilepsies: insights from neuropsychology and neuroimaging. Front Neurol. 2020;11:144. 10.3389/fneur.2020.00144 32210904 PMC7076110

[7] Tosun D , Siddarth P , Toga AW , Hermann B , Caplan R . Effects of childhood absence epilepsy on associations between regional cortical morphometry and aging and cognitive abilities. Hum Brain Mapp. 2011;32(4):580–591. 10.1002/hbm.21045 21391248 PMC3058615

[8] Tondelli M , Vaudano AE , Ruggieri A , Meletti S . Cortical and subcortical brain alterations in juvenile absence epilepsy. NeuroImage Clin. 2016;12:306–311. 10.1016/j.nicl.2016.07.007 27551668 PMC4983643

[9] Jacobs J , LeVan P , Moeller F , Boor R , Stephani U , Gotman J , et al. Hemodynamic changes preceding the interictal EEG spike in patients with focal epilepsy investigated using simultaneous EEG‐fMRI. Neuroimage. 2009;45(4):1220–1231. 10.1016/j.neuroimage.2009.01.014 19349236

[10] Bai X , Vestal M , Berman R , Negishi M , Spann M , Vega C , et al. Dynamic time course of typical childhood absence seizures: EEG, behavior, and functional magnetic resonance imaging. J Neurosci. 2010;30(17):5884–5893. 10.1523/JNEUROSCI.5101-09.2010 20427649 PMC2946206

[11] Killory BD , Bai X , Negishi M , Vega C , Spann MN , Vestal M , et al. Impaired attention and network connectivity in childhood absence epilepsy. Neuroimage. 2011;56(4):2209–2217. 10.1016/j.neuroimage.2011.03.036 21421063 PMC3105167

[12] Guo JN , Kim R , Chen Y , Negishi M , Jhun S , Weiss S , et al. Impaired consciousness in patients with absence seizures investigated by functional MRI, EEG, and behavioural measures: a cross‐sectional study. Lancet Neurol. 2016;15(13):1336–1345. 10.1016/S1474-4422(16)30295-2 27839650 PMC5504428

[13] Wandschneider B , Centeno M , Vollmar C , Symms M , Thompson PJ , Duncan JS , et al. Motor co‐activation in siblings of patients with juvenile myoclonic epilepsy: an imaging endophenotype? Brain. 2014;137(9):2469–2479. 10.1093/brain/awu175 25001494 PMC4132647

[14] Caciagli L , Ratcliffe C , Xiao F , van Graan LA , Trimmel K , Vollmar C , et al. The cognitive phenotype of juvenile absence epilepsy and its heritability: an investigation of patients and unaffected siblings. Epilepsia. 2023;64:2004–2022. 10.1111/epi.17719 PMC10952612 37475704

[15] Caciagli L , Paquola C , He X , Vollmar C , Centeno M , Wandschneider B , et al. Disorganization of language and working memory systems in frontal versus temporal lobe epilepsy. Brain. 2022;146(3):935–953. 10.1093/brain/awac150 PMC9976988 35511160

[16] Lieberman MD , Cunningham WA . Type I and type II error concerns in fMRI research: re‐balancing the scale. Soc Cogn Affect Neurosci. 2009;4(4):423–428. 10.1093/scan/nsp052 20035017 PMC2799956

[17] Caciagli L , Wandschneider B , Xiao F , Vollmar C , Centeno M , Vos SB , et al. Abnormal hippocampal structure and function in juvenile myoclonic epilepsy and unaffected siblings. Brain. 2019;142(9):2670–2687. 10.1093/brain/awz215 31365054 PMC6776114

[18] Poldrack RA , Baker CI , Durnez J , Gorgolewski KJ , Matthews PM , Munafò MR , et al. Scanning the horizon: towards transparent and reproducible neuroimaging research. Nat Rev Neurosci. 2017;18(2):115–126. 10.1038/nrn.2016.167 28053326 PMC6910649

[19] Masur D , Shinnar S , Cnaan A , Shinnar RC , Clark P , Wang J , et al. Pretreatment cognitive deficits and treatment effects on attention in childhood absence epilepsy. Neurology. 2013;81(18):1572–1580. 10.1212/WNL.0b013e3182a9f3ca 24089388 PMC3806916

[20] Xiao F , Caciagli L , Wandschneider B , Joshi B , Vos SB , Hill A , et al. Effect of anti‐seizure medications on functional anatomy of language: a perspective from language functional magnetic resonance imaging. Front Neurosci. 2022;15:1–12. 10.3389/fnins.2021.787272 PMC8908426 35280343

[21] Wandschneider B , Hong SJ , Bernhardt BC , Fadaie F , Vollmar C , Koepp MJ , et al. Developmental MRI markers cosegregate juvenile patients with myoclonic epilepsy and their healthy siblings. Neurology. 2019;93(13):e1272–e1280. 10.1212/WNL.0000000000008173 31467252 PMC7011863

[22] Whelan CD , Altmann A , Botía JA , Jahanshad N , Hibar DP , Absil J , et al. Structural brain abnormalities in the common epilepsies assessed in a worldwide ENIGMA study. Brain. 2018;141(2):391–408. 10.1093/brain/awx341 29365066 PMC5837616

[23] Gordon EM , Chauvin RJ , Van AN , Rajesh A , Nielsen A , Newbold DJ , et al. A somato‐cognitive action network alternates with effector regions in motor cortex. Nature. 2023;617(7960):351–359. 10.1038/s41586-023-05964-2 37076628 PMC10172144

[24] Jensen MA , Huang H , Valencia GO , Klassen BT , van den Boom MA , Kaufmann TJ , et al. A motor association area in the depths of the central sulcus. Nat Neurosci. 2023;26(7):1165–1169. 10.1038/s41593-023-01346-z 37202552 PMC10322697

[25] Ji G j , Fox MD , Morton‐dutton M , Wang Y , Sun J , Hu P , et al. A generalized epilepsy network derived from brain abnormalities and deep brain stimulation. Nat Commun. 2025;16(1):2783. 10.1038/s41467-025-57392-7 40128186 PMC11933423

[26] Badawy RAB , Vogrin SJ , Lai A , Cook MJ . Capturing the epileptic trait: cortical excitability measures in patients and their unaffected siblings. Brain. 2013;136(4):1177–1191. 10.1093/brain/awt047 23485850

[27] Uddin LQ , Supekar K , Menon V . Typical and atypical development of functional human brain networks: insights from resting‐state fMRI. Front Syst Neurosci. 2010;4:1–12. 10.3389/fnsys.2010.00021 20577585 PMC2889680

[28] Goulden N , Khusnulina A , Davis NJ , Bracewell RM , Bokde AL , McNulty JP , et al. NeuroImage the salience network is responsible for switching between the default mode network and the central executive network: replication from DCM. Neuroimage. 2014;99:180–190. 10.1016/j.neuroimage.2014.05.052 24862074

[29] Woermann FG , Sisodiya SM , Free SL , Duncan JS . Quantitative MRI in patients with idiopathic generalized epilepsy. Evidence of widespread cerebral structural changes. Brain. 1998;121(9):1661–1667. 10.1093/brain/121.9.1661 9762955

[30] Verrotti A , Matricardi S , Rinaldi VE , Prezioso G , Coppola G . Neuropsychological impairment in childhood absence epilepsy: review of the literature. J Neurol Sci. 2015;359(1–2):59–66. 10.1016/j.jns.2015.10.035 26671087

[31] Caplan R , Siddarth P , Stahl L , Lanphier E , Vona P , Gurbani S , et al. Childhood absence epilepsy: behavioral, cognitive, and linguistic comorbidities. Epilepsia. 2008;49(11):1838–1846. 10.1111/j.1528-1167.2008.01680.x 18557780

[32] Binding LP , Dasgupta D , Giampiccolo D , Duncan JS , Vos SB . Structure and function of language networks in temporal lobe epilepsy. Epilepsia. 2022;63(5):1025–1040. 10.1111/epi.17204 35184291 PMC9773900

[33] Buckner RL , Andrews‐Hanna JR , Schacter DL . The brain's default network: anatomy, function, and relevance to disease. Ann N Y Acad Sci. 2008;1124:1–38. 10.1196/annals.1440.011 18400922

[34] Thomas Yeo BT , Krienen FM , Sepulcre J , Sabuncu MR , Lashkari D , Hollinshead M , et al. The organization of the human cerebral cortex estimated by intrinsic functional connectivity. J Neurophysiol. 2011;106(3):1125–1165. 10.1152/jn.00338.2011 21653723 PMC3174820

[35] Raichle ME . The brain's default mode network. Annu Rev Neurosci. 2015;38:433–447. 10.1146/annurev-neuro-071013-014030 25938726

[36] Kennedy DP , Redcay E , Courchesne E . Failing to deactivate: resting functional abnormalities in autism. Proc Natl Acad Sci U S A. 2006;103(21):8275–8280. 10.1073/pnas.0600674103 16702548 PMC1472462

[37] Garrity AG , Pearlson GD , McKiernan K , Lloyd D , Kiehl KA , Calhoun VD . Aberrant “default mode” functional connectivity in schizophrenia. Am J Psychiatry. 2007;164:1–8. 17329470 10.1176/ajp.2007.164.3.450

[38] Wandschneider B , Burdett J , Townsend L , Hill A , Thompson PJ , Duncan JS , et al. Effect of topiramate and zonisamide on fMRI cognitive networks. Neurology. 2017;88(12):1165–1171. 10.1212/WNL.0000000000003736 28213372 PMC5373787

[39] Wandschneider B , Stretton J , Sidhu M , Centeno M , Kozák LR , Symms M , et al. Levetiracetam reduces abnormal network activations in temporal lobe epilepsy. Neurology. 2014;83(17):1508–1512. 10.1212/WNL.0000000000000910 25253743 PMC4222853

[40] Haneef Z , Levin HS , Chiang S . Brain graph topology changes associated with anti‐epileptic drug use. Brain Connect. 2015;5(5):284–291. 10.1089/brain.2014.0304 25492633 PMC4490704

[41] Xiao F , Caciagli L , Wandschneider B , Sander JW , Sidhu M , Winston G , et al. Effects of carbamazepine and lamotrigine on functional magnetic resonance imaging cognitive networks. Epilepsia. 2018;59(7):1362–1371. 10.1111/epi.14448 29897625 PMC6216427

[42] Szaflarski JP , Allendorfer JB . Topiramate and its effect on fMRI of language in patients with right or left temporal lobe epilepsy. Epilepsy Behav. 2012;24(1):74–80. 10.1016/j.yebeh.2012.02.022 22481042 PMC3564045

[43] Xiao B , Zhang Y , Wandschneider F , Wang G , Halpin B , Horwitz A , et al. Progressive changes in brain morphology in idiopathic generalised epilepsy. Neurology. 2014;83:2326–2334.

