# Supplementary material for: Syndrome‐specific and familial imaging traits in juvenile absence epilepsy
Source: Epilepsia. 2026 Jan 13;67(4):1887–900. doi: 10.1002/epi.70094 (PMC13075608; doi:10.1002/epi.70094)
Supplement: Supplementary file 1 — Table S1. Group comparisons of functional and structural analysis. Table S2. Correlation analyses of working memory functional magnetic resonance imaging and structural voxel‐based analysis. Figure S1. Working memory task contrasts for each group. [file EPI-67-1887-s001.docx]

**Syndrome-Specific and familial imaging traits in Juvenile Absence Epilepsy**

**Running head**: Imaging traits in juvenile absence epilepsy

Keywords: Sensorimotor system, syndrome-specific, magnetic resonance imaging, endophenotype

Fenglai Xiao^1,2^†, MD, PhD, Lorenzo Caciagli^1,2,3^†, MD, PhD, Luisa Delazer^1,2,4,5^†,Sjoerd Vos^1,2,6,7,8^ PhD, Karin Trimmel^1,2,9^, MD, PhD, Louis Andre Van Graan^1,2^, PhD, Marine Fleury^1,2^, Lawrence Binding^1,2,6,7^, PhD, Davide Giampiccolo^1,2,10,11^, MD, Dominic Heaney^1,2^, MD, PhD, Sanjeev Rajakulendran^1,2,12^, MD, PhD, Maria Centeno^1,2,13^, MD, PhD, Josemir W. Sander^1,2,14^, FMedSci,, John S. Duncan^1,2^, DM, FRCP, Matthias J. Koepp^1,2^*, MD, PhD, Britta Wandschneider ^1,2^*, MD, PhD

† These authors contributed equally to this work as first authors.

* These authors contributed equally to this work as senior authors.

Author affiliations:

*1 Department of Clinical & Experimental Epilepsy, UCL Queen Square Institute of Neurology, London, WC1N 3BG, United Kingdom.*

*2 Chalfont Centre for Epilepsy, Chalfont St Peter, Bucks, SL9 0RJ, United Kingdom.*

*3 Department of Neurology, Inselspital, Sleep-Wake-Epilepsy-Center, Bern University Hospital, University of Bern, Bern, Switzerland*

*4 Epilepsy Center, Department of Neurology, LMU University Hospital, LMU Munich, Germany*

*5 Department of Neurology, Medical University of Innsbruck, 6020 Innsbruck, Austria*

*6 Centre for Medical Image Computing, Departments of Computer Science, Medical Physics, and Biomedical Engineering, UCL, London, WC1E 6BT, United Kingdom.*

*7 Neuroradiological Academic Unit, UCL Queen Square Institute of Neurology,*

*University College London, London, United Kingdom.*

*8 Western Australia National Imaging Facility, The University of Western Australia, Nedlands, Australia*

*9 Department of Neurology, Medical University of Vienna, 1090 Vienna, Austria*

*10 Victor Horsley Department of Neurosurgery, National Hospital for Neurology and Neurosurgery*

*London, WC1N 3BG, United Kingdom*

*11 Institute of Neurosciences, Cleveland Clinic London, London, SW1X 7HY, United Kingdom*

*12 North Middlesex University Hospital, Sterling Way, London N18 1QX, UK.*

*13 Epilepsy Unit, Department of Neurology, Hospital Clínic de Barcelona, 08036 Barcelona, Spain.*

*14 Stichting Epilepsie Instellingen Nederland – (SEIN), Heemstede 2103SW, Netherlands*

[f.xiao@ucl.ac.uk](mailto:f.xiao@ucl.ac.uk), [lorenzo.caciagli@gmail.com](mailto:lorenzo.caciagli@gmail.com), [luisa.delazer@hotmail.com](mailto:luisa.delazer@hotmail.com), [sjoerd.vos@uwa.edu.au](mailto:sjoerd.vos@uwa.edu.au), [k.trimmel@ucl.ac.uk](mailto:k.trimmel@ucl.ac.uk), [louis.graan.12@alumni.ucl.ac.uk](mailto:louis.graan.12@alumni.ucl.ac.uk), [marine.fleury.20@ucl.ac.uk](mailto:marine.fleury.20@ucl.ac.uk), [lawrence.binding.19@ucl.ac.uk](mailto:lawrence.binding.19@ucl.ac.uk), [d.giampiccolo@ucl.ac.uk](mailto:d.giampiccolo@ucl.ac.uk), [d.heaney@ucl.ac.uk](mailto:d.heaney@ucl.ac.uk), [s.rajakulendran@ucl.ac.uk](mailto:s.rajakulendran@ucl.ac.uk), [m.centeno@ucl.ac.uk](mailto:m.centeno@ucl.ac.uk), [l.sander@ucl.ac.uk](mailto:l.sander@ucl.ac.uk), [j.duncan@ucl.ac.uk](mailto:j.duncan@ucl.ac.uk), [m.koepp@ucl.ac.uk](mailto:m.koepp@ucl.ac.uk), [b.wandschneider@ucl.ac.uk](mailto:wandschneider.britta@gmail.com)

Correspondence to: Dr Britta Wandschneider

Department of Clinical and Experimental Epilepsy

UCL Queen Square Institute of Neurology

Queen Square, London, WC1N 3BG

E-mail: [b.wandschneider@ucl.ac.uk](mailto:b.wandschneider@ucl.ac.uk)

Tel: +44 1494 601350

**Supplementary Table 1 (sTable 1): Group comparisons of functional and structural analysis**

|  | **Regions** | **Peak MNI coordinates, x, y, z** | **T, voxels** | **p-value** |
| --- | --- | --- | --- | --- |
| **WORKING MEMORY FMRI** | | | | |
| **JAE < CTR** | | | | |
| 1-Back-minus-0-Back | L precentral and postcentral gyrus | -42, -19, 40 | -3.5, 268 | <0.001 |
|  | L Rolandic Operculum | -42, -25, 16 | -3.5, 78 | <0.001 |
|  | L Precuneus | -30, -40, 10 | -3.7, 378 | <0.001 |
| 2-Back-minus-0-Back | L precentral and postcentral gyrus | -41, -14, 52 | -4.1, 272 | <0.001 |
|  | L supplementary motor area | -5, -9, 51 | -3.2, 71 | <0.001 |
| **JAE > CTR** | | | | |
| 0-Back | L precentral gyrus | -45, -4, 46 | 4.1, 165 | <0.001 |
|  | R precentral gyrus | 60, -7, 25 | 3.95, 100 | <0.001 |
|  | L superior temporal lobe | 48, -19, 7 | 4.43, 51 | <0.001 |
| 2-Back-minus-0-Back | L precentral gyrus | -57, -4, 43 | 4.42, 359 | <0.001 |
|  | L superior temporal | 48, -19, 7 | 3.00, 36 | 0.003 |
| 2-Back-minus-1-Back | Posterior Cingulate cortex | -6, -40, 19 | 4.8, 255 | <0.001* |
| **JAE < SIB** | | | | |
| 1-Back-minus-0-Back | L precentral gyrus | -27, -16, 49 | -4.3, 368 | <0.001 |
|  | L postcentral gyrus | -27, -40, 76 | -4.6, 130 | <0.001 |
|  | R precentral and postcentral gyrus and cingulate gyrus | 15, 5, 34 | -4.8, 518 | <0.001* |
| 2-Back-minus-0-Back | L postcentral gyrus | -18, -34, 61 | -5.1, 888 | <0.001* |
|  | R postcentral gyrus | 18, -34, 61 | -4.2, 157 | <0.001* |
|  | R precentral gyrus | 30, -19, 40 | -3.7, 77 | <0.001 |
| **JAE > SIB** | | | | |
| 2-Back | Posterior cingulate cortex | 0, -43, 19 | 4.63, 139 | <0.001 |
| 2-Back-minus-1-Back | Posterior cingulate cortex | -3, -43, 22 | -4.2, 533 | <0.001 |
|  | L angular gyrus | -51, -73, 31 | -3.3, 74 | <0.001 |
| **STRUCTURAL VOLUME-BASED ANALYSIS** | | | | |
| **JAE < CTR** | L inferior frontal gyrus | -36, 36, -24 | -3.7,719 | <0.001 |
|  | Bilateral motor regions | -4.5, -10.5, 79.5 | -5.1, 6372 | <0.001* |
|  | R medial occipital cortex | 21, -99, -7.5 | -3.7, 618 | <0.001 |
| **JAE > CTR** | L middle cingulate cortex | -7.5, -30, 37.5 | 4.5, 916 | <0.001 |
| **SIB > CTR** | L dorsal middle cingulate cortex | -6, -28.5, 39 | 4.3, 1091 | <0.001 |

CTR = Controls; JAE = Juvenile Absence Epilepsy; L= left; MNI = Montreal Neurological Institute; R= right; SIB = Unaffected siblings of JAE patients. * Also surviving voxel-based FWE-correction (pFWE<0.05, two-tailed).

**Supplementary Table 2 (sTable 2): Correlation analyses of working memory fMRI and structural voxel-based analysis**

| **Working memory fMRI** | **Regions** | **Peak MNI coordinates, x, y, z** | **T, voxels** | **p-value** |
| --- | --- | --- | --- | --- |
| **All participants** |  |  |  |  |
| Lower activation at 0-Back with better executive PC | L precentral gyrus | -45, -13, 61 | -5.3, 134 | <0.001* |
| Lower activation at 1-Back with better executive PC | L precentral gyrus | -48, -10, 58 | -4.4, 61 | <0.001 |
| Lower activation at 2-Back with better executive PC | L precentral gyrus | -45, -10, 58 | -4.4, 95 | <0.001 |
| Higher activation at 1-Back-minus-0-Back contrast with better executive PC | L precentral gyrus | -36, -22, 58 | 4.3, 81 | <0.001 |
| Higher activation at 2-Back-minus-0-Back contrast with better executive PC | L precentral gyrus | -36, -22, 55 | 3.7, 21 | <0.001 |
| Higher activation at 2-Back-minus-1-Back contrast with better executive PC | R superior parietal lobe | 36, -55, 67 | -4.7, 36 | <0.001 |
| **JAE** |  |  |  |  |
| Higher activation at 2-Back-minus-0-Back with longer duration | L postcentral gyrus | -42, -40, 60 | 4.7, 28 | <0.001 |
| Higher activation at 2-Back-minus-0-Back with earlier age of onset | L postcentral gyrus | -45, -43, 58 | -4.8, 30 | <0.001 |
| Higher activation at 2-Back-minus-0-Back with higher drug load score | R paracentral gyrus and supplementary motor area | 3, -25, 67 | 5.4, 25 | <0.001 |
| **Structural Volume-based analysis** |  |  |  |  |
| **All participants** |  |  |  |  |
| Increased structural volume with higher Executive function PC | L precentral gyrus | -38, 3, 46 | 4.3, 2601 | <0.001 |
|  | R precentral gyrus | 10, -22, 72 | 4.3, 3152 | <0.001 |
|  | R superior frontal gyrus | 6, 18, 58 | 4.5, 1024 | <0.001 |
|  | L cuneus | -15, -81, 2 | 4.3, 1476 | <0.001 |
| Increased structural volume with higher language function PC | L middle and inferior frontal gyri | -33, 33, 16 | 4.2, 5292 | <0.001 |
|  | R supplementary motor area | 15, 3, 76 | 4.1, 1572 | <0.001 |
|  | Bilateral caudate nuclei | 12, 0, 0 | 4.3, 3240 | <0.001 |

JAE = Juvenile Absence Epilepsy; L= left; MNI = Montreal Neurological Institute; PC = Principal Component; R= right; * Also surviving voxel-based FWE-correction (pFWE<0.05, two-tailed).

**Supplementary Figure (sFigure): Working memory task contrasts for each group**

﻿Brain renders show voxel-based activation (warm colours) and deactivation (cold colours) of task contrasts in people with JAE, unaffected siblings (SIB) and healthy controls (CTR), as derived from one-sample t-tests. Cortical activation for the three different task contrasts: Motor cortex and supplementary motor deactivation, bilateral frontal and parietal activation. With increasing cognitive demand (2-Back-minus-1-Back), people with JAE showed less activation/deactivation, respectively. The maps are displayed at an uncorrected threshold (*p*<0.0001, cluster extent threshold of 20 voxels).

**
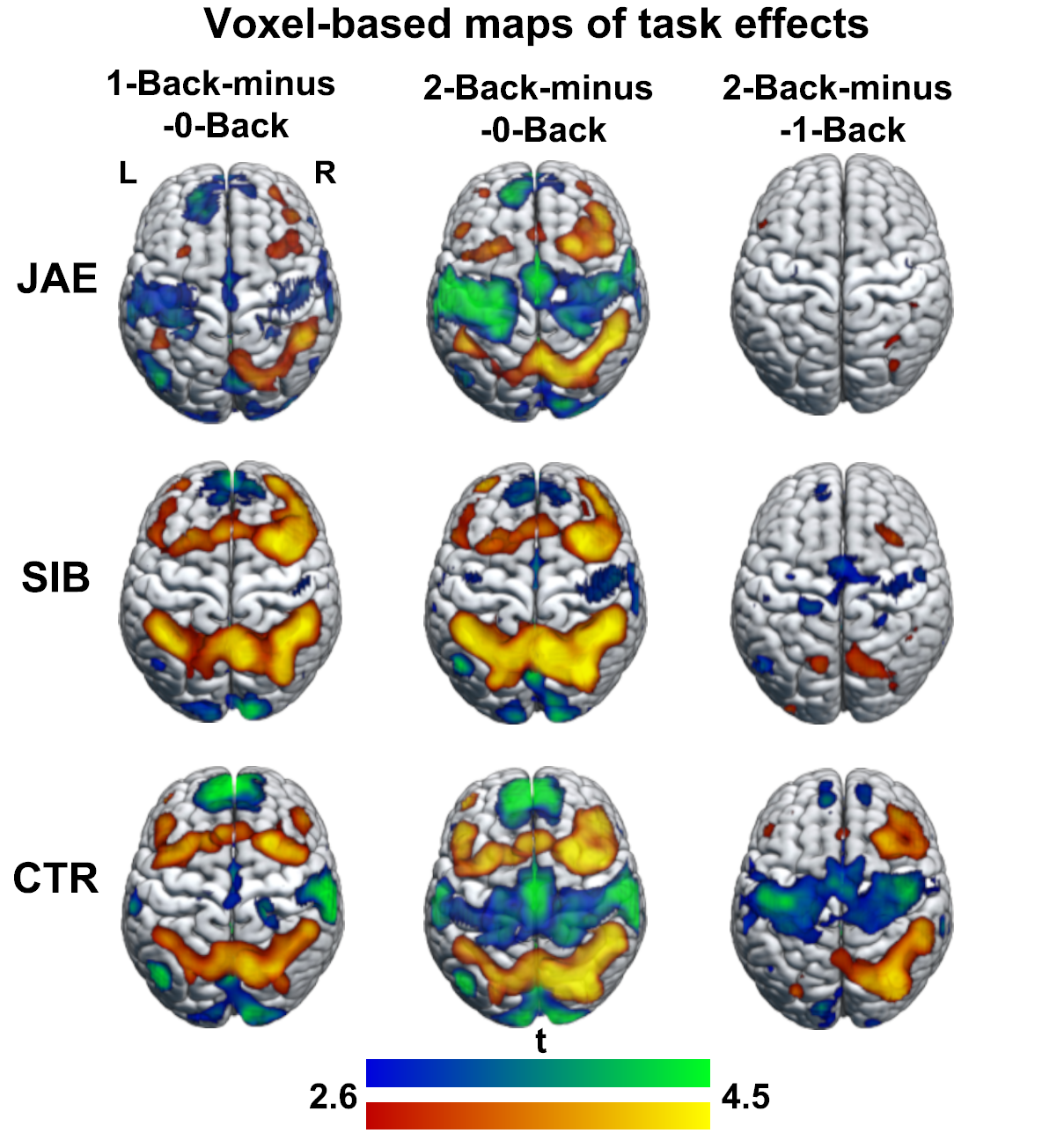
**
